# Influence of Freeze-Drying and Oven-Drying Post Blanching on the Nutrient Composition of the Edible Insect *Ruspolia differens*

**DOI:** 10.3390/insects8030102

**Published:** 2017-09-16

**Authors:** Forkwa Tengweh Fombong, Mik Van Der Borght, Jozef Vanden Broeck

**Affiliations:** 1 Molecular Developmental Physiology and Signal Transduction lab, Division of Animal Physiology and Neurobiology, Department of Biology, University of Leuven, 3000 Leuven, Belgium; tengweh.fombong@kuleuven.be (F.T.F.); jozef.vandenbroeck@kuleuven.be (J.V.B.); 2 Lab4Food, Technology Cluster Bioengineering Technology, Department of Microbial & Molecular Systems, University of Leuven, 2440 Geel, Belgium

**Keywords:** Kenya, entomophagy, feed, food, grasshopper, lipid, preservation method, micronutrient

## Abstract

The longhorn grasshopper, *Ruspolia differens* (Serville), plays an important role as a food source across Sub-Saharan Africa, where it is consumed as a delicacy in both rural and urban areas. The effect of two drying methods (freeze-drying and oven-drying), employed after blanching, on the proximate, fatty acid and mineral composition of the two most common morphs was determined. *Ruspolia differens* grasshoppers were harvested in Uganda and Kenya from wild swarms during the rainy periods of November–December 2016. Based on cuticular coloration, we identified three morphs, green, brown and purple, which occurred at a ratio of 65:33:2, respectively. Results indicated that these insects have a high lipid content of 36%, as well as significant protein levels ranging between 33% and 46% dry matter. Oleic acid (44%) and palmitic acid (28%) were the two most abundant fatty acids; while the presence of arachidonic acid (0.6%) and docosahexaenoic acid (0.21%) suggests that *Ruspolia differens* is also a source of polyunsaturated fatty acids. The observed amino acid profile showed similar trends in all morphs, and all essential amino acids were present. Calcium (896–1035 mg/100 g), potassium (779–816 mg/100 g) and phosphorus (652–685 mg/100 g) were quite high among the minerals. The presence of the trace elements iron (217–220 mg/100 g), zinc (14.2–14.6 mg/100 g), manganese (7.4–8.3 mg/100 g) and copper (1.66 mg/100 g) suggests that inclusion of these grasshoppers in human diets may aid in combatting micronutrient deficiencies. Oven-drying *Ruspolia differens* delivered the same nutritional quality as freeze-drying. Hence, both drying approaches can be adequately used to formulate insect-based food products without noticeable nutritional changes.

## 1. Introduction

The global surge in demand for inexpensive alternative and sustainable protein sources has led the FAO to advocate the consumption of insects [1,2]. Amongst other issues, this international organization is strongly encouraging entomophagy, the intentional eating of insects by humans.

Worldwide, nearly 842 million people (12% of the global population) were unable to meet their dietary energy requirements in 2015–2016. The majority of these people (827 million) live in developing regions, where the prevalence of undernourishment was at 14.3% in 2015. Africa remains the region with the highest occurrence of malnutrition, with Sub-Saharan Africa’s figures at 24.8% [3]. In Sub-Saharan Africa (SSA), entomophagy based on harvesting from the wild is practiced, but many challenges, such as seasonal availability, sustainability concerns, pathogen risks and high perishability of the harvested insects, are associated with this practice. SSA is a region of the world where hunger is expected to increase in the next two decades unless drastic measures are taken to mitigate food insecurity [4]. To respond not just to the rapid population growth, but also to other pressing challenges including climate change and rising volatility of food prices, SSA needs to accelerate its agricultural productivity and efficacy without delay [5]. In this context, FAO recommends the use of sustainable diets that have a low environmental impact and contribute to food and nutrition security for present and future generations. Recently, a high-profile review outlined the importance of insects in assuring food and feed security [6]. Therefore, the FAO has promoted the consumption of insects as a sustainable protein and food source [6].

Globally, more than 2000 species of insects are used as food by at least two billion people. Many insects are comparable to conventional livestock meat regarding nutritional content [7], but with the added benefit of converting a far higher ratio of feed to high-quality protein for human consumption [6]. The latter translates into a much better ecological footprint of mass-produced insects compared to beef and other livestock, with significantly lower greenhouse gas emissions, water and land requirements [6].

Studies of their nutritional potential have shown that grasshoppers contain relatively more high-quality protein than commercially available animal sources used for poultry feed [8]. Grasshoppers have a high reproductive potential with short life cycles of 2–3 months [9,10]. In a recent review [11], the authors suggested that some grasshopper species are an excellent source of nutrients and possess several health-related benefits when consumed.

Consumption of the locally-occurring *R*. *differens* (Orthoptera; Tettigoniidae) is a major part of the food culture in several regions of East-Africa. This nondestructive species is spread across southern, central and eastern Africa. It is also referred to as ‘nsenene’ and is the most consumed grasshopper in Uganda, parts of western Kenya and Tanzania [12,13,14]. For many households, trade in this edible insect is a primary source of income and contributes considerably to improvements in livelihood [12]. Currently, *R. differens* is harvested in the wild during the swarming seasons, which coincide with the rainy seasons of March–April and October–December. Being nocturnal, these ‘bush crickets’ are collected at night, when lights attract them [13,15]. The species displays cuticular color polymorphism, with green and brown morphs being the most common, but as many as six color forms exist [16]. This color polymorphism has not been studied to date; however, previous sources point to a genetic attribute [17]. In Tanzania, purple morphs appear sparingly in swarms, but anecdotally are thought to possess higher ostentatious value due to their color and better taste. These edible grasshoppers (together with palm weevils and termites) are amongst a select few insects eaten in Africa due to both lack of other food sources and because they are delicious [1].

The swarming behavior of *Ruspolia* observed in several localities has revealed the broad diet of these insects comprising grains and grasses of the ubiquitous Poaceae family and their tendency to live together in high densities. Their nutrient content is therefore considered to be both high and variable [6], which indicates tremendous potential for this edible insect to combat human nutritional deficiencies. However, preservation steps are crucial to their processing in order to extend their shelf-life beyond 24 h [18].

Given that many insects are only seasonally available, they are typically preserved for later consumption by drying in the sun, over ashes or in the oven [1]. In Kenya, tettigoniids are traditionally consumed as a snack, either fresh, toasted or sun-dried, depending on the season [15]. While the storage of insects under deeply frozen conditions ensures food quality and safety, drying allows storage at ambient temperatures.

Drying is a traditional method of food preservation used worldwide. Besides, sun-drying, commercial-drying methods, such as drum, evaporation, spray and freeze-drying, are used to preserve a vast array of vegetable- and animal-based foodstuffs. A typical drying process begins with a heating step (e.g., cooking or blanching) to inactivate most of the microorganisms and enzymes present, then the application of dry heat to remove any water contained in the foodstuff [19]. Two commonly-employed drying methods in the food industry are oven-drying and freeze-drying. For tilapia fish, for instance, the preferred method is freeze-drying, as it is known to cause the least damage to proteins [20]. Considering the high capital and running cost associated with the freeze-drying process, alternate drying methods (i.e., sun- or oven-drying) are more frequently used in developing nations for preserving insects. Research on other animals (e.g., tilapia fish) has shown that different drying methods had different effects on the nutritional composition, which was attributed to the chemical and physical changes caused by exposure to heating or freezing [20]. It seems logical that such effects would influence the nutrient composition of insects, as well. Nonetheless, investigations into the effect of different drying processes on the nutritional properties of insects are limited.

In Kenya, the emergence and growth of the middle class, combined with increasing Westernization, is driving the demand for more sophisticated processed food products, with greater variety and improved nutritional quality. Kenyan tastes and preferences are also increasingly influenced by foreign travel and wider access to global brands via the growth of modern grocery retailing [21]. To convince consumers to accept insect products from different processing (drying) methods, an investigation of their effects on the nutritional constituents is required.

Most research on *R. differens* from Kenya has been on the nutritional analyses of the green and brown morph only [13]. The nutritional properties of the purple morph are yet to be established. Furthermore, the amino acid profile of this popular grasshopper remains unknown. The influence of processing methods , i.e., toasting and solar drying, on the in vitro protein digestibility and vitamin content of green and brown grasshoppers consumed in Kenya has been studied [15]. However, the authors did not investigate the influence of the cooking (toasting) and drying method (sun-drying) on the proximate, fatty acid or mineral contents of *R. differens*. Furthermore, only approximate values were given for temperatures used for sun-drying. Consequently, a study that evaluates drying effects based on accurate measurements and includes a broader range of nutritional parameters is necessary. Oven- and freeze-drying remain the most popular methods of preserving *R. differens*. Surprisingly, research that investigates the effect of these two drying methods on the nutritional composition of this African tettigoniid has not been carried out.

Therefore, the aim of this study was to compare the nutritional properties of green, brown and purple grasshopper morphs following two popular drying methods (i.e., oven-drying and freeze-drying). If the methods of drying were found to have little impact on the nutritional composition (i.e., the proximate, fatty acid, amino acid and mineral content) of grasshoppers, then they could be used in novel insect-based food formulations, irrespective of the method by which they were preserved.

## 2. Materials and Methods

### 2.1. Sample Acquisition and Preparation

During the swarming season of October–November 2016, approximately 20 kg of grasshoppers were harvested from the wild from the cities of Masaka and Kampala in Uganda and Busia and Kisumu in Kenya. Grasshoppers were pooled together in specially-designed cages and transported live to our laboratory at the Food Technology department in Jaramogi Oginga Odinga University of Science and Technology (JOOUST) Bondo, Kenya. Upon arrival, they were sorted based on color (morph), before being blanched in boiling water for 5 min, drained and allowed to cool and placed into zip-lock freezer bags and immediately frozen at −20 °C. The frozen samples were then transported in an ice-packed cooler box to the Food Science department of Jomo Kenyatta University of Agriculture and Technology (JKUAT), Nairobi, for drying. They were then either freeze-dried (FD) or oven-dried (OD), as described below.

### 2.2. Drying Methods

The freeze-drying process was performed using the freeze-dryer (CHRIST-ALPHA-LDPLUS-101541, Martin Christ, Osterode am Harz, Germany). Blanched green, brown and purple grasshoppers were frozen at −30 °C for 20 min and then freeze-dried in a vacuum in two steps: main drying (−50 °C at 0.040 bars) for 48 h and final drying (−55 °C at 0.021 bars) for another 48 h.

Blanched green and brown grasshoppers were oven-dried using a laboratory oven (Memmert UF 110, Memmert, Schwabach, Germany) at 60 °C for 24 h. Purple morphs were not oven-dried because they were not collected in sufficient amounts.

Dried samples were ground into powder using a two-speed Waring laboratory blender, (Camlab, Over, UK). Samples were transported to Belgium and stored for a month in the dark in sealed plastic bottles prior to analysis. Compositional analyses of samples were performed in triplicate unless otherwise stated.

### 2.3. Chemical Analyses

#### 2.3.1. Proximate Composition

Moisture content was analyzed using a forced-air oven (UF 110, Memmert, Schwabach, Germany) at 105 °C for 24 h according to [22].

Crude protein content was determined by the Kjeldahl method [23] using a steam distillation apparatus (Vapodest 20, Gerhardt, Königswinter, Germany). The method was verified using acetanilide as the reference standard; the method blank was also included.

Protein content = nitrogen × 6.25 (where 6.25 = protein conversion factor).

Crude fat content was obtained by the Soxhlet method of Nielsen [24] using petroleum ether as the solvent. The solvent was then removed using a rotary evaporator (Büchi, R-200) at 50 °C.

Ash content of the insect samples was determined gravimetrically [25] using a muffle furnace (B 180, Nabertherm, Lilienthal, Germany) overnight at 550 °C. After the determination of the ash content, the ashes were collected in a low-density polyethylene container and stored for mineral analysis.

Chitin content was measured gravimetrically after deproteinization using 1 M NaOH and subsequent demineralization with 1 M HCl using the procedures outlined by Liu et al. [26].

Nitrogen-free extract (NFE) was calculated as: 

100 − (crude proteins + crude lipids + ash + fibers) [27].

Energy content was estimated using the formula: 

[(crude proteins × 17) + (crude lipids × 37) + (NFE × 17)] [28].

#### 2.3.2. Amino Acid Analysis

It has been suggested that diet and, by extension, drying method have little or no effect on the amino acid composition of insects [29]. We therefore decided to perform such analysis only on the FD samples. Furthermore, the close similarity in the values of the protein content of different colored morphs further confirmed the redundancy in repeating the analysis on OD samples.

Before determining the amino acid profile of blanched and FD insect specimens, 25 mg of the samples were subjected to acid hydrolysis using 10 mL of 6 M HCl in 20-mL test tubes. The method described by Hewitson et al. [30] was employed to determine amino acids.

During the acid hydrolysis, asparagine (Asn) and glutamine (Gln) were converted into aspartic acid (Asp) and glutamic acid (Glu), respectively. The UPLC separation of these amino acids was performed on an Acquity UPLC (Waters, Milford, MA, USA), consisting of a PDA detector, column heater, sample manager, binary solvent delivery system and an AccQ·TagTM Ultra column (2.1 i.d. × 100 mm; Waters). Sample derivatization was achieved using the Waters AccQ·Tag Ultra Chemistry Package. Gradient elution was applied according to Waters AccQ·Tag Ultra method (AccQ·Tag Ultra Eluent A Concentrate (10-times diluted) (Waters); AccQ·Tag Ultra Eluent B (Waters), as well as a flow rate of 0.7 mL·min^−1^ and column temperature of 60 °C. Data reprocessing was done using Empower 2 software (Waters, Milford, MA, USA).

#### 2.3.3. Fatty Acid Analysis

Fatty acid methyl esters (FAMEs) were prepared from the lipid samples by esterification in a methanolic KOH solution (0.500 M) with the addition of a 20% BF_3_-methanol solution (Sigma-Aldrich, St. Louis, MO, USA) according to Joseph and Ackman [31]. The fatty acid composition was determined with an Agilent 7820A-5977E GC-MSD (Agilent Technologies, Santa Clara, CA, USA) using the settings in Table 1 below:

#### 2.3.4. Mineral and Trace Elemental Analysis

To determine the mineral composition, the ashes obtained during the determination of the ash content were dissolved in 65% HNO_3_ (VWR Chemicals, Fontenay-sous-Bois, France) and subsequently diluted ten-fold to an appropriate concentration (*i.e.*, depending on the mineral and the calibration curve) [32]. Calibration curves were prepared using standard solutions from certified stock solutions containing 1000 ppm of the elements investigated (Chem Lab, Zedelgem, Belgium). The content of the investigated elements was determined by inductively-coupled plasma optical emission spectrometry (ICP-OES) measurements (Optima 4300™ DV ICP-OES, Perkin Elmer Wellesley MA, USA). All samples were analyzed in duplicate using the following conditions specified in Table 2 below:

### 2.4. Statistical Analysis

To elucidate the effect of drying method (post blanching) on the nutritional composition of the brown and green grasshopper morphs, a Mann–Whitney U-test (alpha = 0.05) was performed on the mean values of OD and FD grasshopper samples. The statistical package used was GraphPad Prism Version 5.00 for Windows (GraphPad Software, La Jolla, CA, USA).

## 3. Results and Discussion

### 3.1. Proximate Composition

The FD materials had a moisture content of 4.55%, 4.91% and 4.24%, for the green, brown and purple-colored morphs, respectively. The final water content for the green and brown OD samples was 4.33% and 4.50%, respectively.

The purple sample was not included in the statistical analyses, as the sample was too small to obtain significant amounts for both drying methods; as such, only FD purple samples were analyzed.

The changes in moisture content, crude fat, crude protein, chitin fibers and ash are depicted in Table 3. In general, neither drying method seems to have had any significant effect on the proximate composition of the grasshoppers.

We found that apart from the purple morph, in which the protein, fat and ash content differed, there was little variation (*p* > 0.99) in the green and brown grasshoppers across both drying methods. These observations are in agreement with Kinyuru et al. [13], who reported similar values and found no difference in protein content and only slight variations in other parameters. The overall variation could also have arisen from the insects’ diets since they were captured at different locations and pooled together. Since they were all caught in the adult stage, the stage of development could not have accounted for such variation, but the sex and reproductive state of insects have been shown to affect nutrient composition [28].

The results for energy content, ash and chitin presented here are consistent with the findings of Rumpold and Schluter [33], who in a review compiled the proximate composition of 51 orthopterans (see Table 3) from the literature.

The moisture content for the insect morphs has been reported in other studies to vary between 66% and 71% [13]. In our case, we obtained average water contents of 53.72 g/100 g fresh weight before drying. Use of different drying times and temperatures would yield different values of moisture for the same species. Adult insects tend to have less moisture than their nymph counterparts, and this trend was evident, as all samples evaluated were adults and had moisture contents lower than expected for grasshoppers [11]. The low moisture content of the fresh grasshoppers when compared with more conventional meat, such as chicken and beef, would imply a higher content of dry matter and, by extension, this means that *R. differens* constitutes a denser source of nutrients, since a relatively larger portion of its weight is represented by nutrients than other animal food sources. The insect samples were dried to moisture contents between 4.0% and 4.5%, a range carefully chosen to extend the shelf storability of the products, as well as to optimize fat extraction using the Soxhlet procedure.

Fat is known to exude with moisture evaporation during oven drying, which increases the effect of lipid and fatty acid losses. This phenomenon has been observed with OD fish in comparison to other preservation methods, as outlined by Chukwu [20]. However, the drying method had no significant effect on the crude fat content in our case. The absence of fat reduction during oven drying in our study is explained by the lower temperature used (being 60 °C, compared to 110 °C) in the preparation of dried tilapia fish [20].

Overall, *R. differens* showed higher crude fat contents (35.5%) than most orthopterans (13.41%), although this was compensated by lower average protein content (47.75 % for OD and 46.41% for FD) in comparison to several other insects of this order (61.32%) [33]. In a recent review, data compiled by Aman et al. [11] reveal that *R. differens* has the highest fat and lowest protein content that so far has been reported in the order of orthoptera. The high lipid content of the mean value of *R. differens* morphs (35.50%) accounts for the insects’ palatability when fried or roasted, as mentioned by other authors [34]. This value is lower than the 46.2–48.2% obtained by other researchers for the same species [13].

The lipid contents were higher than those of chicken, fish and unprocessed milk, but similar to that of raw chicken egg [35]. When compared to beef or fish, these insects had high lipid contents and are therefore also good energy sources. Indeed, lipids are necessary for food because they increase palatability and retain the flavor of food, as well as enhancing vitamin A, D, E and K levels [36,37].

The higher fat content of the FD purple morph compared to the other color morphs explains why the purple grasshoppers are anecdotally considered to be more delicious.

The protein content of most insect species is very high, with many ranging from 60%–85% [2,38]. Orthopterans (crickets, grasshoppers and locusts) averaged 60% protein content, the highest among all edible insect orders compared to the isopteran (termites) with just 35% [33]. The average protein content of *R. differens* was found to be 46.41–47.7%, in agreement with similar studies on this species (43.1–44.3%) [13]. *R. differens* tends to contain midrange levels of protein relative to other edible insects. According to WHO/FAO [39], the requirement for food to be labeled ‘high in protein’ is a 10 g/100 g edible portion. This limit, like for most insects, is far exceeded by *Ruspolia* proteins, and this edible insect can thus be considered a good protein source.

The observed high protein and fat contents, which comprise more than 75% of the dry mass, justify the cultural perception of high nutritional value attributed to *Ruspolia differens.*

Insects are known to contain significant amounts of fiber, and Finke [40] suggested that the fiber in insects is predominantly composed of chitin. Chitin is a major component of the insect cuticle, which is covalently bound to catechol compounds and sclerotin-like proteins [30]. Chitin is present only in the insects’ exoskeleton and is expected to be present in relatively small amounts. Rumpold and Schlüter [33] reported 9.55% as the average fiber content for grasshoppers. This fiber value (predominantly chitin) is close to the mean values (13.4% and 11.33%) for either drying method obtained in the current study. As before, no significant differences were observed between morphs, but a slight influence of drying mode was noticed. However, much lower values were reported by Kinyuru et al. [13] for the same species. *R. differens*’ high chitin content might, therefore, present potential value to both the food and pharmaceutical industries [26].

Average ash content after oven and freeze drying was 4.66% and 4.79%, respectively, which was almost two-fold more than what has been previously reported for the same species (2.7%) [13]. This difference could be explained by the use of different analytical methods. Higher amounts of minerals were also observed as shown in Table 5, but were nonetheless consistent with the average for 51 orthopterans compiled from the literature [33]. Higher ash values of 8.55% and 9.36% in grasshoppers have also been mentioned in other studies [11,33].

Nitrogen-free extract (NFE) levels, largely representing carbohydrates (but not chitin), are usually low in insects, which explains the dearth of information on the carbohydrate content of insects [27]. In the current study, NFE levels were low (0.7–1.99%), but were highest in the green morphs (1.39–3.97%). The average NFE values obtained for each drying method (i.e., 0.7% and 2.0% for OD and FD, respectively) were significantly lower than previously reported for most orthoptera, being approximately 13% [33]. Ramos et al. [41] obtained similarly low values for NFE in the house cricket, *Acheta domestica.* In contrast, extremely high values of up to 63.20% have been reported for the grasshopper *Zonocerus variegatus* [42].

Due to their high fat content, the average energy content (539 and 519 Kcal/g) for this study estimated by calculation [28] was greater than the mean reported for insects. The above statement is true for other lipid-rich insects, in particular caterpillars, palm weevil larvae and termites [43]. The values mentioned above obtained for proximate composition in our study were more consistent with those obtained for a related Ugandan species, *Ruspolia nitidula* [18].

When data are expressed on a dry matter basis, the sum of proximates should exceed the expected level of >95 g/100 g edible portion [44]. This interval was true for the sum of proximates calculated in the current study.

### 3.2. Fatty Acid Composition

The fatty acid composition of the samples examined is shown in Table 4 below. These fatty acids are mainly stored in the insect’s fat body and comprise more than 90% of the total lipid content of the fat body [45]. Very similar values were obtained for all fatty acids irrespective of the drying method.

Oleic (44%), palmitic (28%) and linoleic (14%) acids were the major fatty acid components of *R. differens* contributing up to 86% of the total fatty acids present; a trend that was also observed by Kinyuru et al. [13] for the same species. For FD and OD samples, respectively, the main saturated fatty acids (SFA) were palmitic (C16:0, 28.2% and 27.8%) and stearic acid (C18:0, 7.88% and 8.45%), while the most dominant unsaturated fatty acids (UFA) were oleic (C18:1, 44.3% and 44.0%) and linoleic acid (C18:2, 14.0% and 14.1%). These values were again consistent with other studies [43,45,46,47].

The present study revealed that the samples studied were rich in polyunsaturated fatty acids (PUFA), especially linolenic and linoleic acids. Previous studies [13] did not detect lauric, arachidonic or EPA fatty acids in their samples, as they occur in low proportions and are not present in easily quantifiable amounts. PUFA/SFA ratios lower than 0.33 are not desirable as they likely lead to atherogenesis, whereas PUFA/SFA ratios greater than 0.8 are associated with desirable levels of cholesterol and reduced risk of coronary heart diseases [48]. With a mean PUFA/SFA ratio of 0.44, *R. differens* was found to be significantly higher in SFA than PUFA, though still above the cut-off point for triggering undesirable effects.

Another health index associated with fatty acids is that of the omega-6/omega-3 fatty acid ratio (n6:n3), with a ratio of 3:1 considered optimal [49]. The current study gave an n6:n3 ratio of 9:1 on average, which is regarded as high. Lower ratios of omega-6/omega-3 fatty acids are desirable for reducing the risk for an array of chronic diseases of high occurrence in both developed and developing countries [48]. Previous research revealed significant variation in n6:n3 ratios, due to omega-3 fatty acid differences [50], thus confounding comparisons with existing literature. Essential fatty acids (EFA), which include linoleic and alpha-linolenic acids, were present in appreciable quantities (15.42% and 15.60%) for FD and OD samples, respectively.

Finke [28,45] concluded that, for a given insect species and developmental stage, the fatty acid composition is affected by environmental factors such as temperature, light, and humidity. These influences may also explain the variations observed in this study.

### 3.3. Mineral Composition

Table 5 depicts the mineral composition of the *Ruspolia* grasshoppers. Again, there were no significant differences (*p* > 0.88) between drying methods, although high standard deviations were obtained for the average drying values of some minerals, indicating variability, which has been reported by other researchers, as well [51,52], and attributed to a small sample size or contamination. A trend evident in Table 5 was the higher mineral content in FD relative to OD samples; except for Zn (14.6 and 14.2 mg/100g) and Cu (1.66 mg/100g for both). However, the morph type had a bigger influence than did the drying method (*p* > 0.79). The insects were high in most macro minerals, as well as trace minerals; which is true for most edible insects [28,42,53]. With the exception of sodium (Na), the mineral values obtained in this study were higher than values previously reported [13] for this grasshopper species.

Average calcium (Ca) levels were high, being 895.7 and 1035 mg/100 g dry matter (DM) for OD and FD, respectively; i.e., values well below the recommended daily intake (RDI) for adult humans (1300 mg) [39,54]. According to WHO/FAO [39], foods containing Ca levels above 240 mg/100 g edible portion are considered to be ‘high in calcium’. These results suggest this edible insect could serve as an alternative source of calcium, especially for people who are lactose intolerant or allergic to soy. This is entirely in contrast to statements made by other researchers that insects are low in Ca [28,45,52,55]. The highest value previously reported for Ca in an edible insect was 2010 mg/100 g in the housefly *Musca domestica* (Linnaeus), although some orthopteran species have been found to contain high Ca values (1290 mg/100 g in *Acheta domesticus* (Linnaeus)), as well [33]. This could be attributed to the high Ca content of their gut [41].

Potassium (K) was the next most abundant mineral (779 and 816 mg/100 g DM for OD and FD, respectively), and although levels were found to be very high compared to those reported for other edible insects [33], they did not meet the adult RDI of 4700 mg [54]. These values were less than half those obtained for *Zonocerus variegatus* (Linnaeus) (2030 mg) [56], which are by far the highest recorded K values amongst the grasshopper family.

The Mg content was also elevated at 145 and 161 mg/100 g DM for OD and FD, respectively, compared with previously reported data for *R. differens* (33.1–33.9 mg/100g) [13]. Comparable values for other orthopterans, such as the cricket, *Acheta domesticus*, have been reported in the literature [56]. Adult RDI for Mg lies within 220–260 mg; thus, consuming about 200 g (100 fresh grasshoppers) in a day will surpass the Mg RDI.

Like for most edible insects, the samples evaluated in this study were also found to be high in phosphorus 652.31–685.9 mg/100 g DM for OD and FD, respectively. It has been suggested in the literature that a Ca:P ratio of 1:1 to 1:2 is acceptable in food and feed for most vertebrates [57]. This ratio incorporates the one obtained in this study (1:1.5). Thus, the inclusion of *R. differens* in feed for other animals could balance the Ca:P ratio required for most diets.

The trace minerals were also present in good amounts (Table 5), particularly Fe and Zn, which concurs with other reports [8,53,58]. Consumption of mineral-rich insects could help mitigate Fe and Zn deficiencies, which are prevalent in developing countries [53]. That Fe was the most prominent of the trace minerals was also true for Kinyuru et al. [13], though much higher Fe levels of between 216 and 220 mg/100 g were measured in the current study. The RDI of Fe for female adults stands between 20 and 59 mg, depending on bioavailability [59]. When compared to the Fe levels present in a variety of red meats (1.1–3.3 mg/100 g) [60], *R. differens* is a superabundant source of Fe, although the bioavailability of Fe is not known.

Very similar zinc (Zn) concentrations (being 14.6 and 14.2 mg/100 g) were observed for the two drying methods, and these were comparable to levels reported previously [13,52]. The RDI of Zn for adults is between 4.9 and 7.0 mg (moderate bioavailability) [59]; as such, either FD or OD preparation of *R. differens* would provide a relatively rich source of Zn.

Another trace metal, manganese (Mn) was present, at levels ranging between 7.4 and 8.3 mg/100 g, i.e., levels above the RDI of 1.8–2.6 mg [59]. *Ruspolia* was found to be a significant source of selenium in the brown morphs within 40–50 µg/100 g, which exceeds the adult RDI of 26–36 µg.

The samples investigated in this study yielded promising mineral compositions, i.e., levels surpassed those of conventional meats [60]. The compositional difference observed between different color morphs could be ascribed to environmental factors [40].

### 3.4. Amino Acid Composition

Table 6 comprises the amino acid content of the FD grasshoppers; with the amino acids commonly found in proteins being identified in these samples. During acid hydrolysis, asparagine (Asn) and glutamine (Gln) were converted into aspartic acid (Asp) and glutamic acid (Glu), respectively.

No significant difference in amino acid content was observed between the two methods as was to be expected given the similar overall protein content. With the exception of methionine and cysteine, the essential amino acids were present at concentrations that met, and surpassed, the levels recommended for humans by the WHO/FAO. Glutamic acid (+ glutamine), alanine and aspartic acid (+ asparagine) were the most abundant amino acids. Defoliart [58] showed that insect proteins are low in methionine and cysteine. This was true in the current study, however, the opposing belief that insect proteins are high in threonine and lysine was not observed. Nonetheless, not all insects are the same in their nutritional contributions.

Protein quality and nutritional value are determined by the amino acid composition and digestibility of the protein fraction of foods [6,44]. Lysine and threonine are indispensable since they are not transaminated, and their deamination is irreversible. The *R. differens* amino acid profile was found to be high in leucine, lysine and threonine. In the predominantly cereal-based diet common in developing nations, lysine and threonine are particularly limiting. Therefore, the inclusion of this insect species into the staple diets of these nations would be expected to significantly improve the nutritional status of their population.

Another crucial parameter for the correct determination of protein quality is the ratio of essential I and nonessential (N) amino acids. According to the FAO/WHO criteria, E/(E + N) should be about 40% with E/N = 0.6 [54]. FAO/WHO/European food safety authority (EFSA) dietary criteria state that adults should consume 0.66 g/kg of body weight of protein, daily [61]. In the current study, an E/N of 0.61 and E/(E + N) value of 38% indicate that the amino acid composition of *R. differens* satisfies these criteria.

## 4. Conclusions

Oven drying blanched *R differens* delivers the same nutritional (proximate, mineral and fatty acid) quality and composition as freeze drying; either drying method could be used to preserve nutrient properties. Differences observed in nutritional composition were attributed to the different morphs, rather than to the method of drying employed. Importantly, all of the essential amino acids required by humans were found to be present.

Insect samples from both drying approaches provided good sources of macronutrients, as well as minerals, and are therefore deemed suitable as alternative or complementary food sources to alleviate undernutrition, especially among vulnerable groups (i.e., women and children) in developing countries. The rare purple morph yielded similar nutritional values as the green and brown morphs, but their slightly elevated fat content may explain anecdotal perceptions that this phenotype is ‘tastier’.

## Figures and Tables

**Table 1:** GC-MS parameter settings.

Parameter	Settings
*Volume sample injected*	1.0 µL
*Ratio split injector*	10:1
*Carrier gas*	Helium
* Temperature*	40 °C
*Pressure*	12 psi
*Capillary column*	Sigma-Aldrich SLB-IL60
*Stationary phase (SP)*	1,12-di(tripropylphosphonium)dodecane-*bis*-(trifluoromethylsulfonyl)imide
*Length*	30 m
*Diameter*	0.25 mm
*Thickness SP*	0.2 µm
*Oven*	Start: 4 minutes at 40 °C | during analyses: increase of 5°C/minute | end: 280 °C
*Ion source MS*	Electron impact
*Scan type MS*	Single ion monitoring
*Software*	MassHunter

Methyl tricosanoate was used as the internal standard to determine the fatty acid composition.

**Table 2:** ICP-OES working conditions‘ settings.

Sampler		Spectrometer	
**Parameter**	**Setting**	**Parameter**	**Setting**
Plasma conditions	Same for each element	Pulsed gas flow	Normal
Type Aerosol	Wet	Spectral profiling	No
Start nebulizer	Directly	Resolution	Fixed (normal)
Sample flow (mL/min)	1.5	Reading time (s)	Automatic
Plasma sight (all)	Radial	Break time (s)	30
Plasma sight (element)	Axial	Replicas (#)	3
Source delay (s)	30	Software	WinLab 32
Flush time (s)	10		

**Table 3:** Proximate composition of oven-dried (OD) and freeze-dried (FD) green, brown and purple grasshoppers (in % on a dry matter basis). Each value expresses the mean ± SD of triplicate determinations. NFE, nitrogen-free extract.

Samples	Green OD	Brown OD	Mean OD	Green FD	Brown FD	Purple FD ^a^	Mean FD ^b^	Orthoptera ^c^
Moisture content	4.33	4.50	4.42	4.55	4.91	4.24	4.42	Not available
Crude fat	34.95 ± 1.01	36.11 ± 0.34	35.53 ± 0.82	33.28 ± 0.38	36.84 ± 0.62	38.8 ± 0.40	35.56 ± 1.82	**13.41**
Crude protein	45.53 ± 0.84	52.90 ± 0.86	47.7 ± 3.09	44.99 ± 0.74	47.83 ± 1.62	50.50 ± 0.35	46.41 ± 2.01	**61.32**
Chitin	14.86 ± 0.58	14.22 ± 0.73	13.4 ± 1.13	9.79 ± 1.06	10.81 ± 1.17	11.2 ± 1.11	11.33 ± 0.74	**9.55**
Ash	3.93 ± 0.03	5.38 ± 0.00	4.66 ± 1.03	4.92 ± 0.07	4.66 ± 0.13	4.32 ± 0.32	4.79 ± 0.18	**3.85**
NFE	1.39	0.01	0.7 ± 0.98	3.97	0.01	0.01	1.99 ± 2.80	**12.98**
Energy (Kcal/g)	510	539	524 ± 20.6	501	537	566	519 ± 25	**426.25**

^a^ Due to its small sample size, the purple-colored grasshoppers were only freeze-dried; ^b^ mean value excluded the purple morph and was therefore not considered during statistical analyses; ^c^ Orthoptera average values as compiled by Rumpold and Schluter [33].

**Table 4:** Fatty acid composition (% of total fatty acids) of FD and OD grasshoppers. Values are expressed as the mean of triplicates ± SD; *: mean ± SD of combined color morphs.

Fatty Acid	Type	Green FD	Brown FD	Mean FD *	Brown OD	Green OD	Mean OD *
Decanoic Acid (C10:0)	SFA	0.07 ± 0.04	0.07 ± 0.03	0.07 ± 0.01	0.06 ± 0.01	0.07 ± 0.04	0.07 ± 0.01
Lauric acid (C12:0)	SFA	0.19 ± 0.01	0.19 ± 0.01	0.19 ± 0.01	0.17 ± 0.01	0.16 ± 0.02	0.17 ± 0.01
Myristic Acid (C14:0)	SFA	1.14 ± 0.10	1.13 ± 0.07	1.14 ± 0.01	1.11 ± 0.06	1.08 ± 0.09	1.10 ± 0.02
Kyriologic acid (C14:1)	MUFA	0.07 ± 0.04	0.08 ± 0.03	0.08 ± 0.01	0.07 ± 0.01	0.07 ± 0.04	0.07 ± 0.01
Pentadecanoic acid (C15:0)	SFA	0.11 ± 0.04	0.12 ± 0.03	0.12 ± 0.01	0.11 ± 0.01	0.12 ± 0.04	0.12 ± 0.01
Palmitic acid (C16:0)	SFA	28.2 ± 0.79	28.1 ± 0.65	28.2 ± 0.11	27.3 ± 0.70	28.2 ± 1.09	27.8 ± 0.62
Palmitoleic acid (C16:1)	MUFA	1.71 ± 0.16	1.68 ± 0.09	1.70 ± 0.02	1.64 ± 0.11	1.62 ± 0.11	1.63 ± 0.01
Heptadecanoic acid (C17:0)	SFA	0.15 ± 0.04	0.15 ± 0.03	0.15 ± 0.01	0.14 ± 0.01	0.15 ± 0.05	0.15 ± 0.01
Stearic acid (C18:0)	SFA	7.92 ± 0.67	7.84 ± 0.43	7.88 ± 0.06	8.60 ± 0.63	8.30 ± 0.46	8.45 ± 0.21
Oleic Acid (C18:1)	MUFA	44.4 ± 1.86	44.3 ± 0.72	44.3 ± 0.11	43.7 ± 1.03	44.3 ± 1.48	44.0 ± 0.42
Linoleic acid (C18:2) [n6]	PUFA	14.0 ± 1.50	14.0 ± 1.41	14.0 ± 0.04	14.4 ± 1.63	13.9 ± 0.67	14.1 ± 0.32
Linolenic Acid (C18:3) [n3]	PUFA	1.39 ± 0.14	1.44 ± 1.08	1.42 ± 0.04	1.47 ± 1.16	1.43±0.13	1.45 ± 0.03
Arachidonic acid (C20:4) [n6]	PUFA	0.39 ± 0.01	0.72 ± 0.56	0.56 ± 0.23	0.93 ± 0.43	0.44 ± 0.02	0.69 ± 0.35
EPA (C20:5) [n3]	PUFA	0.14 ± 0.09	0.23 ± 0.14	0.19 ± 0.06	0.30 ± 0.17	0.15 ± 0.10	0.23 ± 0.11
TOTAL SFA		37.82	37.58	37.7	37.52	38.08	37.8
TOTAL MUFA		46.19	46.02	46.11	45.43	46.00	45.72
TOTAL PUFA		15.99	16.4	16.20	17.05	15.92	16.49
TOTAL UFA		62.18	62.42	62.3	62.48	61.92	62.2
PUFA/SFA ratio		0.42	0.44	0.43	0.45	0.42	0.44
Total n6		14.46	14.73	14.60	15.28	14.34	14.81
Total n3		1.53	1.67	1.60	1.77	1.58	1.68
n6/n3 ratio		9.45	8.82	9.12	8.63	9.08	8.84
EFA		15.39	15.44	15.42	15.87	15.33	15.6

EPA = methyl-5, 8, 11, 14, 17-eicosapentaenoic acid; n6 = omega-6 fatty acid; n3 = omega-3 fatty acid; PUFA = polyunsaturated fatty acid; SFA = saturated fatty acid; MUFA = monounsaturated fatty acid; UFA = unsaturated fatty acid; EFA = essential fatty acid = [C18:2 + C18:3].

**Table 5:** Mineral composition (mg/100 g dry matter) of OD and FD grasshoppers. Results are expressed as the mean ± SD; (n = 2). <LOQ = below the limit of quantification.

Mineral	OB	OG	Mean OD	FG	FB	Mean FD
Na	50.79 ± 0.02	57.2 ± 0.01	54.01 ± 4.55	78 ± 0.01	60.2 ± 0.01	69.1 ± 12.64
K	834.4 ± 0.04	724.0 ± 0.06	779.19 ± 78.0	806 ± 0.08	826.5 ± 0.06	816.4 ± 14.27
Ca	1124 ± 0.05	967.6 ± 0.06	895.67 ± 323	1023 ± 0.08	1047 ± 0.10	1034.7 ± 17.18
Mg	168.5 ± 0.01	123.0 ± 0.01	145.75 ± 32.1	160 ± 0.01	161.8 ± 0.01	161.0 ± 1.06
Zn	14.24 ± 0.01	15.0 ± 0.01	14.63 ± 0.56	13 ± 0.01	15.2 ± 0.01	14.2 ± 1.46
Fe	258.7 ± 0.01	174.4 ± 0.01	216.56 ± 59.6	217 ± 0.05	222.8 ± 0.01	220.1 ± 3.83
P	693.9 ± 0.03	610.7 ± 0.06	652.31 ± 58.8	680 ± 0.05	692.1 ± 0.06	685.9 ± 8.73
Cu	1.67 ± 0.01	1.6 ± 0.01	1.66 ± 0.01	1 ± 0.01	1.8 ± 0.01	1.66 ± 0.23
Mn	8.77 ± 0.01	6.0 ± 0.01	7.40 ± 1.94	8 ± 0.01	8.4 ± 0.01	8.29 ± 0.21
Se	0.05 ± 0.01	<LOQ	<LOQ	<LOQ	0.04 ± 0.02	<LOQ

**Table 6:** Average amino acid content of the FD green, brown and purple morph (mg/g protein). Results are expressed as the mean ± SD; (n = 3).

Amino acid	Green	Brown	Purple	WHO/FAO+
His	24.66 ± 0.12	25.80 ± 0.36	27.00 ± 0.43	15.0
Ser	48.05 ± 0.90	48.92 ± 0.63	50.59 ± 0.20	--
Arg	57.31 ± 3.49	55.04 ± 3.04	61.77 ± 3.99	--
Gly	60.72 ± 0.90	64.86 ± 0.79	59.38 ± 0.03	--
Asp	96.79 ± 3.20	92.95 ± 3.14	95.00 ± 3.67	--
Glu	123.3 ± 4.71	124.7 ± 4.22	122.6 ± 4.10	--
Thr	41.75 ± 0.58	42.98 ± 0.38	42.78 ± 0.45	23
Ala	117.0 ± 4.96	117.3 ± 4.46	104.73 ± 4.56	--
Pro	62.61 ± 1.65	64.63 ± 1.74	61.34 ± 1.64	--
Cys	5.18 ± 2.13	2.86 ± 2.53	6.88 ± 2.69	6.0
Lys	54.77 ± 3.89	53.80 ± 3.26	53.16 ± 3.29	45
Tyr	52.53 ± 4.46	49.57 ± 4.16	55.19 ± 4.15	--
Met	6.99 ± 8.76	1.39 ± 8.26	13.83 ± 8.82	16
Val	65.75 ± 0.89	67.06 ± 0.40	63.48 ± 0.07	39
Ile	47.61 ± 1.04	49.17 ± 1.20	46.91 ± 1.81	30
Leu	92.48 ± 1.84	95.22 ± 1.09	90.30 ± 1.52	59
Phe	33.79 ± 0.62	35.48 ± 0.83	36.88 ± 0.52	--
Trp	8.66 ± 0.28	8.26 ± 0.14	9.38 ± 0.32	6.0
E	376	379	384	
N	624	621	617	
E/N	0.60	0.61	0.62	
E + N	1000	1000	1001	
E/(E + N)	0.38	0.38	0.38	

E = essential amino acid; N = nonessential amino acids; + amino acid requirements in humans [39]; -- data not available.
